# Integrative Multiomics Analysis Reveals the Ameliorative Effects of *Astragalus membranaceus* Extract on Metabolic Dysfunction-Associated Steatotic Liver Disease

**DOI:** 10.3390/molecules31071120

**Published:** 2026-03-28

**Authors:** Jiayi An, Yi Li, Zunhan Zhang, Yaru Chang, Guanxiu Xiao

**Affiliations:** 1 College of Biological Sciences and Technology, Yili Normal University, Yining 835000, China; ajy1129@126.com (J.A.);; 2 College of Life Sciences, Henan Normal University, Xinxiang 453007, China

**Keywords:** Astragalus extract, MASLD, gut microbiota, metabolomics, network pharmacology

## Abstract

Metabolic dysfunction-associated steatotic liver disease (MASLD) is a growing global health burden, yet effective therapeutic options remain limited. This study investigated the protective mechanisms of *Astragalus membranous* extract (AM) against high-fat diet (HFD)-induced MAFLD in mice using an integrated strategy combining network pharmacology, hepatic metabolomics, and 16S rRNA sequencing. UPLC–Q-Orbitrap–MS/MS identified 37 major constituents in AM, mainly phenolic acids and flavonoids. Iristectorin A, isorhamnetin, ononin, and rhamnocitrin were identified as key candidate compounds due to their relatively high abundance and confirmation as absorbed constituents in vivo. Network pharmacology and molecular docking indicated favorable interactions with hub targets (TNF, EGFR, and AKT1; binding energies < −5.0 kcal/mol) and highlighted the involvement of the AGE–RAGE signaling pathway and inflammation- and lipid metabolism-related processes. In vivo, AM significantly attenuated HFD-induced weight gain, decreased serum ALT and AST levels, and reduced hepatic lipid deposition. AM also alleviated oxidative stress by lowering malondialdehyde (MDA) and increasing superoxide dismutase (SOD) activity, while suppressing hepatic IL-1β and IL-6. Moreover, AM improved gut microbial homeostasis by restoring α-diversity and enriching beneficial genera, including Akkermansia and Bacteroides. Hepatic metabolomics further showed that AM partially normalized lipid metabolic disturbances, particularly glycerophospholipid and sphingolipid metabolism. Collectively, these results suggest that AM mitigates MASLD via a multi-component, multi-target mechanism, potentially through modulation of AGE–RAGE-associated inflammatory signaling and the gut–liver axis, supporting its development as a functional food-derived candidate for metabolic liver disorders.

## 1. Introduction

Metabolic dysfunction-associated fatty liver disease (MASLD) is a prevalent multisystemic disorder characterized by excessive lipid accumulation in hepatocytes, occurring independently of significant alcohol consumption [1,2]. Globally, MASLD affects approximately 38% of the adult population and has become a primary driver of chronic liver disease [3]. Without timely intervention, MASLD can progress from simple steatosis to metabolic dysfunction-associated steatohepatitis (MASH), hepatic fibrosis, cirrhosis, and even hepatocellular carcinoma [4,5,6]. Despite its high prevalence and severe prognosis, there are currently no regulatory-approved pharmacological therapies specifically for MASLD [7]. Existing clinical options often face challenges regarding limited long-term efficacy and potential safety concerns, necessitating the search for novel, safe, and effective alternative interventions [8,9]. In this context, Traditional Chinese Medicine (TCM) offers a promising strategy due to its multi-component, multi-target properties and generally favorable safety profile [10].

*Astragalus membranaceus* (AM), known as “Huangqi” in China, has been a cornerstone of TCM for over 2000 years, traditionally used to treat metabolic and inflammatory conditions [11,12,13]. Modern pharmacological research has demonstrated that AM contains a diverse array of bioactive constituents, including saponins, polysaccharides, and flavonoids, which exhibit potent anti-inflammatory, antioxidant, and hepatoprotective activities [14,15]. Recent studies have indicated that specific AM-derived monomers and extracts can mitigate liver injury by inhibiting oxidative stress and modulating autophagy [16]. Furthermore, emerging evidence suggests that AM can regulate the intestinal microenvironment to alleviate metabolic disorders [17]. However, the systematic material basis and the integrated molecular mechanisms by which AM extract ameliorates MASLD remain to be fully elucidated.

The “gut–liver axis” describes the bidirectional crosstalk between the gastrointestinal tract and the liver, mediated by metabolites, microbial antigens, and nutrients [18,19]. Gut microbiota dysbiosis plays a pivotal role in the pathogenesis of MASLD by increasing intestinal permeability and promoting hepatic inflammation. It is well established that gut microbiota dysbiosis plays a pivotal role in the pathogenesis of by increasing intestinal permeability and altering short-chain fatty acids (SCFAs) and bile acid profiles [20]. Consequently, remodeling the gut microbiota and maintaining intestinal homeostasis have emerged as key therapeutic targets. To decode the complex interactions between herbal extracts and the host, integrative “multiomics” approaches are increasingly employed. Network pharmacology identifies potential “drug–target–disease” interactions at the protein level, while metabolomics characterizes the global shifts in small-molecule endogenous metabolites [21].

In the present study, an integrated approach encompassing network pharmacology, hepatic metabolomics, and 16S rRNA sequencing was employed to systematically investigate the therapeutic potential of AM against high-fat diet (HFD)-induced MASLD in mice. We first characterized the chemical profile of AM and validated its efficacy in mitigating hepatic steatosis and inflammation. Subsequently, we elucidated its regulatory impact on gut microbiota homeostasis and hepatic lipid metabolism, with a specific focus on glycerophospholipid and sphingolipid pathways. To further decode the molecular basis of these effects, bioinformatics analysis and molecular docking were conducted to identify hub therapeutic targets and their potential interactions with AM-derived bioactive compounds. This research provides new mechanistic insights and a scientific basis supporting the potential application of AM as a functional food constituent for MAFLD management

## 2. Results

### 2.1. Chemical Profiling of AM by UPLC-Q-Orbitrap-MS/MS Analysis

**Figure 1:**
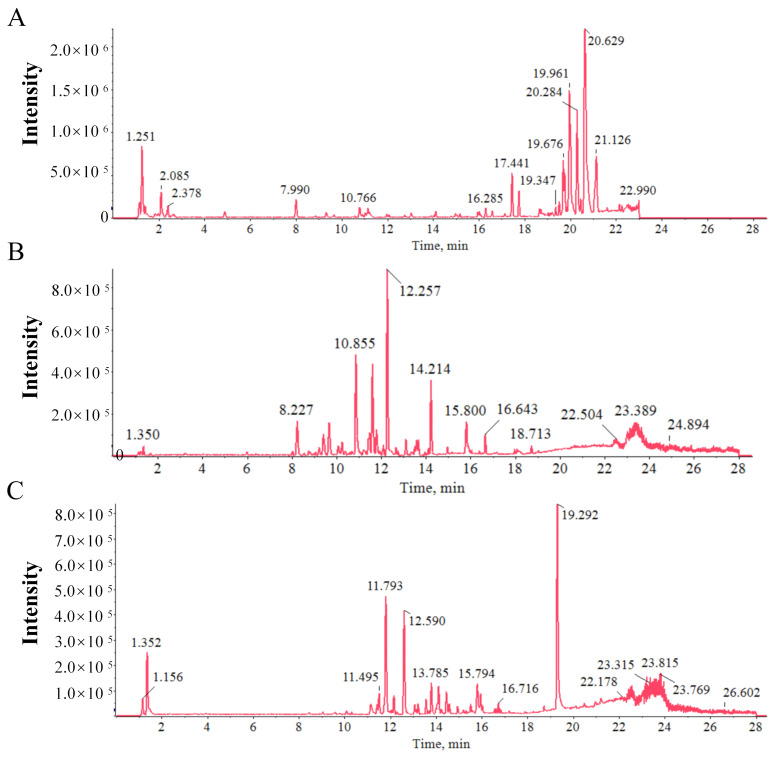
Total ion current derived from a UPLC-Q-orbitrap-MS/MS chromatogram in negative ion mode. (**A**) Blank serum specimen. (**B**) AM solution. (**C**) Serum specimen obtained from mice following oral administration of AM. Note: cps represents counts per second.

The chemical constituents of AM were first systematically characterized using UPLC–Q–Orbitrap–MS/MS operated in both positive and negative electrospray ionization (ESI) modes. Compound identification was achieved by comprehensively analyzing retention times, theoretical and measured accurate masses, MS/MS fragmentation patterns, previously reported literature data, and comparisons with available reference standards. A total of 37 chemical components were tentatively identified in AM, including 15 flavonoids, 10 triterpenoid saponins, 1 organic acid, 2 nucleobases, 2 amino acids, and 7 others (Figure 1). Among these constituents, ten compounds were detected in plasma after administration, indicating their potential bioavailability in vivo. The total ion chromatogram (TIC) in negative ion modes of AM is displayed in Figure 1, and the detailed information on the representative compounds is summarized in Table 1.

### 2.2. Network Pharmacology and Molecular Docking Analysis

**Figure 2:**
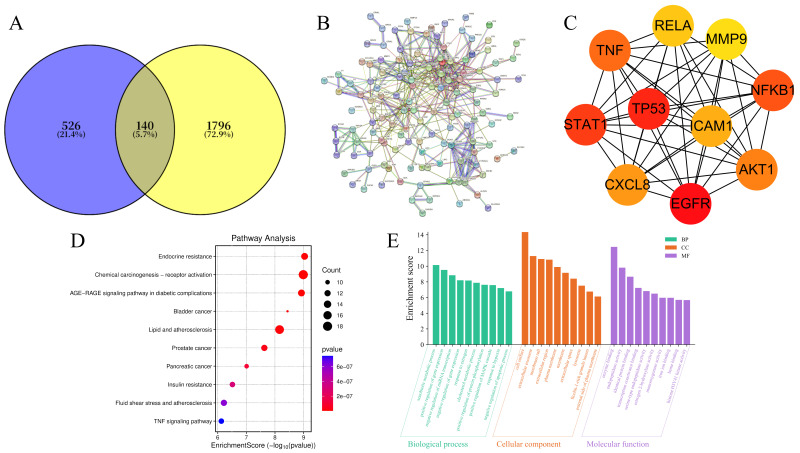
Network pharmacology analysis of the mechanism of AM in MASLD. (**A**) Venn diagram depicting the intersection between MASLD-related genes and AM-targets. (**B**) PPI network established based on the identified targets. (**C**) Hub gene interaction network extracted from the PPI network. (**D**) Pathway analysis identifying significant signaling pathways associated with MASLD. (**E**) GO functional enrichment analysis bar chart presenting the major enriched terms for biological process (BP), cellular component (CC), and molecular function (MF).

To systematically explore the therapeutic mechanism of AM against MASLD, network pharmacology was integrated with molecular docking. Based on the screening of bioactive constituents (Table 1), the top 10 compounds were identified as the primary active components of AM. Using public databases, a total of 346 potential targets associated with these components were predicted, and a comprehensive “compound-target” network was constructed (Figure S1). Among these, Iristectorin A, Isorhamnetin, Ononin, and Rhamnocitrin emerged as key bioactive compounds due to their high degree of connectivity. Intersection analysis between AM-related targets and MASLD-associated disease targets revealed 140 overlapping genes (Figure 2A), which were considered potential therapeutic targets. To further elucidate the core regulatory hub, a Protein–Protein Interaction (PPI) network was established (Figure 2B). Using the CytoHubba plugin within Cytoscape (v3.7.2), the top 10 hub targets were identified—AKT1, TNF, TP53, EGFR, STAT3, MMP9, NFKB1, CXCL8, ICAM1, and RELA (Figure 2C)—suggesting their pivotal roles in the pharmacological effects of AM. Subsequently, Gene Ontology (GO) and KEGG pathway enrichment analyses were performed to decode the functional implications of these targets (Figure 2D,E). The results indicated that these targets are predominantly involved in biological processes such as cell cycle regulation, DNA repair, and immune response, localized within the nucleus and cell membrane. Notably, KEGG enrichment highlighted the significant involvement of the AGE-RAGE signaling pathway, lipid metabolism, and inflammation-related cascades, all of which are strongly associated with the complex pathological processes of MASLD.

**Figure 3:**
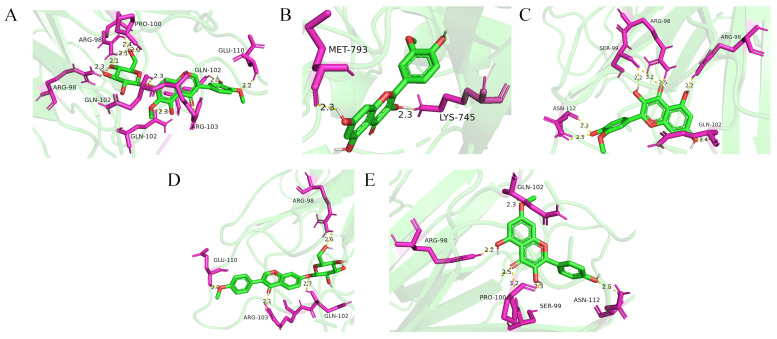
Representative outcomes of molecular docking between the screened compounds and the selected targets. (**A**) Binding mode of Iristectorin A with TNF; (**B**) binding mode of Isorhamnetin with EGFR; (**C**) binding mode of Isorhamnetin with TNF; (**D**) binding mode of Ononin with TNF; (**E**) binding mode of Rhamnocitrin with TNF.

To validate the interactions between the identified key compounds and hub targets, molecular docking was performed. As summarized in Table S1, the binding affinities for the majority of the ligand–receptor complexes were lower than −5.0 kcal/mol, indicating strong thermodynamic stability and high binding affinity. Representative docking configurations (Figure 3) further demonstrated that the key bioactive compounds of AM could fit precisely into the active pockets of their respective targets through hydrogen bonding and hydrophobic interactions. These findings provide computational evidence that AM exerts a multi-compound and multi-target therapeutic effect in the management of MASLD.

### 2.3. AM Alleviated the Pathological Condition of MASLD Mice

**Figure 4:**
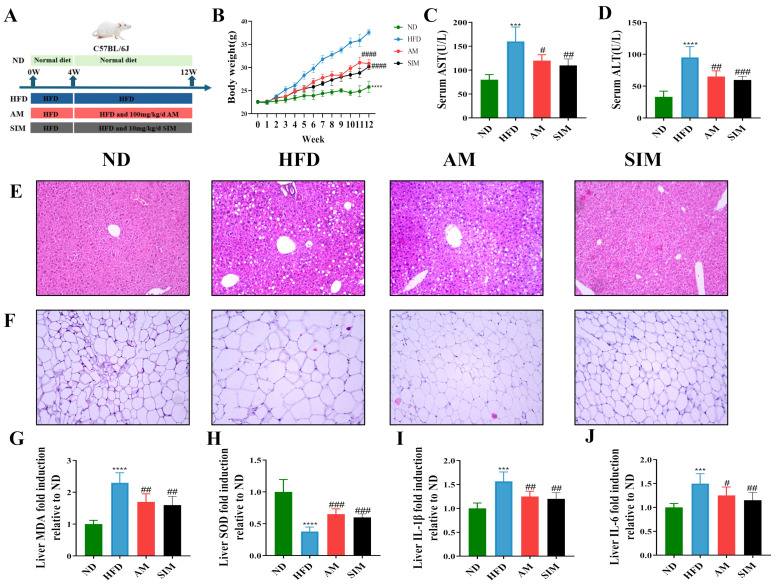
Effects of AM on MASLD-related pathological indicators in HFD-induced mice. (**A**) In the animal experiment procedure, the ND group is denoted in green, the Model group in blue, the AM group in red, and the Sim group in black. (**B**) Body mass; (**C**) serum ALT level; (**D**) serum AST level; (**E**) representative H&E-stained liver sections (magnification: 200×, scale bar = 100 μm); (**F**) representative H&E-stained images of epididymal white adipose tissue (eWAT) (magnification: 200×, scale bar = 100 μm); (**G**) liver MDA level; (**H**) liver SOD level; (**I**) liver IL-1β level; and (**J**) liver IL-6 level of the four groups. *** *p* < 0.001, **** *p* < 0.01 when compared with the ND group; # *p* < 0.05, ## *p* < 0.01, ### *p* < 0.001, #### *p* < 0.0001 when compared with the HFD group when compared with the HFD group.

The experimental timeline for the animal study is schematically illustrated in Figure 2A. To evaluate the anti-obesity effects of AM, body weight was monitored weekly. As depicted in Figure 2B, HFD feeding induced a substantial and progressive increase in body weight compared with the control group; however, this weight gain was significantly curtailed by AM supplementation. Concurrently, serum biochemical analysis revealed that AM treatment markedly reversed the HFD-induced elevation of ALT and AST levels (Figure 4C,D), suggesting AM showed a certain protective effect on liver function in MASLD mice. H&E staining further corroborated the hepatoprotective efficacy of AM. The HE staining results showed that the HFD group had a large number of fat vacuoles compared to the ND group, while the AM group displayed a reduced production of fat vacuoles to some extent (Figure 4E). Furthermore, H&E staining of white adipose tissue revealed that AM effectively suppressed HFD-induced adipocyte hypertrophy, resulting in significantly smaller adipocyte diameters compared to the model group (Figure 4G). These findings demonstrated that AM limited the accumulation of liver fat and effectively improved adipose tissue morphology. In MASLD mice induced by an HFD. Oxidative stress and inflammation are commonly associated with MASLD, a finding consistent with the results of this experiment. The AM reduced the accumulation of lipid peroxidation product MDA and mitigated the abnormal reduction in the antioxidant enzyme SOD in the liver (Figure 4H,I). Additionally, inflammatory cytokines IL-1β and IL-6 in the liver were similarly suppressed (Figure 4I,J). Post hoc power analysis and Cohen’s *d* calculations revealed large effect sizes and high statistical power, indicating that the observed differences are robust despite the limited sample size. Collectively, these results demonstrate that AM effectively alleviates HFD-induced MASLD by remodeling adipose and hepatic morphology and dampening oxidative and inflammatory responses. H&E staining revealed hepatic steatosis, hepatocellular ballooning, and mild inflammatory cell infiltration.

### 2.4. AM Improved Gut Microbiota Dysbiosis in NAFLD Mice

**Figure 5:**
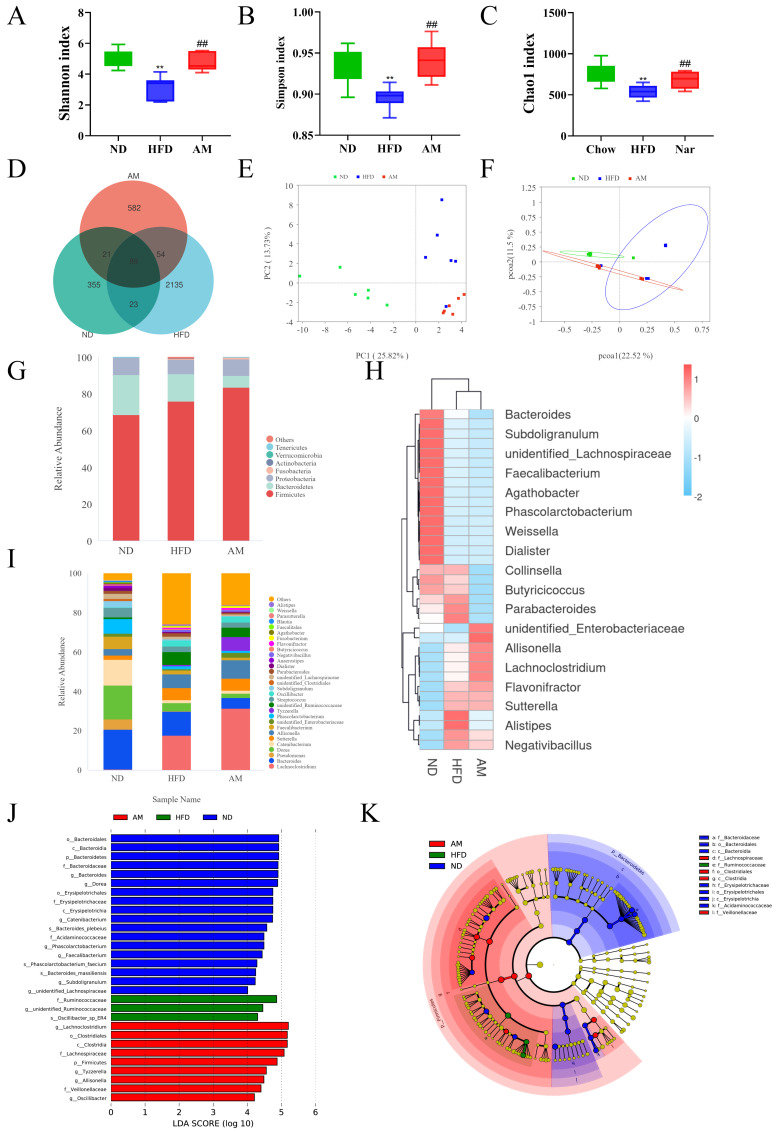
Influences of AM on the gut microbiota in HFD-induced MASLD mice. (**A**) Shannon diversity index. (**B**) Simpson’s diversity index. (**C**) Chao 1 richness index. (**D**) Venn diagram showing shared and unique OTUs among groups. (**E**) PCA of the gut microbiota. (**F**) PCoA of the gut microbiota. (**G**) Relative abundance of gut microbiota at the phylum level. (**H**) Relative abundance of gut microbiota at the genus level. (**I**) Heatmap illustrating the genus-level microbial composition. (**J**) LDA score distribution of differentially abundant taxa. (**K**) LEfSe analysis. Data are presented as mean ± SD. Statistical significance is indicated as follows: ** *p* < 0.01 when compared to the chow group; ## *p* < 0.01 when compared to the HFD group.

Alpha diversity analysis based on *t*-test was performed to evaluate the differences in the microbial community among groups (Figure 5A–C). The Shannon and Simpson indices of the HFD group were significantly decreased compared with the ND group (*p* < 0.05), indicating that high-fat diet feeding reduced the diversity of intestinal flora. In contrast, the Shannon and Simpson indices of the AM group were significantly increased compared with those of the HFD group, suggesting that AM treatment effectively restored microbial richness and diversity in MASLD mice. The Venn diagram (Figure 5D) revealed a total of 3259 bacterial operational taxonomic units (OTUs) were identified, with 355 OTUs in the ND group and 2135 OTUs in the HFD group. Among them, 89 OTUs were shared across all three groups, indicating differential microbial compositions. As shown in Figure 3E,F, both principal coordinate analysis (PCA) and Principal Coordinate Analysis (PCoA) analyses demonstrated that the intestinal microbiota profiles of the ND and HFD groups were significantly different. After AM administration, the microbiota composition of the HFD mice shifted toward that of the ND group, indicating that AM intervention remodeled the disturbed microbial community. At the phylum level (Figure 5G), the gut microbiota of HFD-fed mice was dominated by *Firmicutes* and *Bacteroidetes*, with a significant increase in the *Firmicutes*/*Bacteroidetes* (F/B) ratio compared with the ND group. AM supplementation decreased the F/B ratio toward normal levels, suggesting improved microbial balance. At the genus level (Figure 5H–I), the relative abundances of *Bacteroides*, *Faecalibacterium*, and *Agathobacter* were reduced in the HFD group, whereas *Lachnospiraceae*, *Ruminococcaceae*, and *Collinsella* were elevated. AM treatment reversed many of these alterations, restoring beneficial taxa and reducing potentially pathogenic ones. Biomarkers with an LDA score > 4 were screened and displayed as an LDA histogram (Figure 5J) and a LefSe evolutionary branching diagram (Figure 5K). Analysis showed that *Lachnospiraceae* and *Collinsella* were significantly enriched in the HFD group, whereas *Bacteroides*, *Faecalibacterium*, and *Akkermansia* were dominant in the AM group. These results indicate that AM could reshape HFD-induced changes in the relative abundance of intestinal flora and restore microbial homeostasis in MASLD mice.

### 2.5. AM Improved Hepatic Metabolic Disorder in MASLD Mice

**Figure 6:**
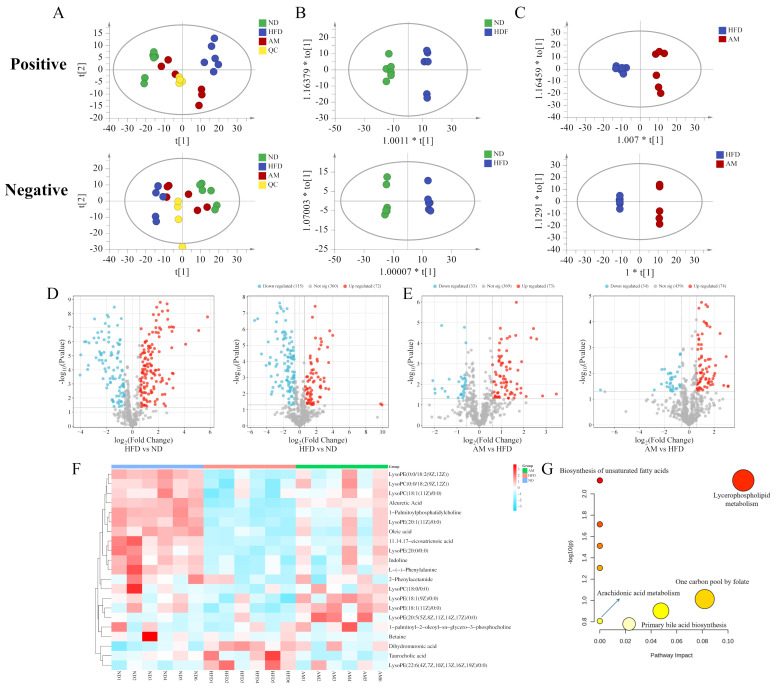
Influence of AM on the Liver Metabolic Profile in HFD-Induced MASLD Mice. (**A**) PCA score plots in positive and negative ion modes for the ND, HFD, AM, and quality control (QC) groups. (**B**,**C**) OPLS-DA score plots comparing the ND group with the HFD group (**B**) and the HFD group with the AM group. (**D**,**E**) Volcano plots depicting differential metabolites between the HFD and ND groups (**D**) and between the AM and HFD groups (**E**) in both ion modes. (**F**) Heatmap of representative differential metabolites among groups. (**G**) KEGG pathway enrichment analysis based on the identified differential metabolites.

To elucidate the alterations in endogenous hepatic metabolites and the therapeutic impact of AM, a non-targeted metabolomics approach was employed. Initially, PCA, an unsupervised multivariate statistical method, was performed to visualize the metabolic trajectories among the ND, HFD, and AM-treated groups. As illustrated in the PCA score plot (Figure 6A), distinct clustering patterns were observed, indicating significant metabolic separation. The HFD group exhibited a marked deviation from the ND group, reflecting substantial metabolic perturbations induced by a high-fat diet. Notably, the AM-treated group showed a clear trend of migration toward the ND cluster, suggesting that AM intervention partially restored the hepatic metabolic profile of MASLD mice toward a homeostatic state. Furthermore, the tight aggregation of quality control (QC) samples demonstrated the high stability and reproducibility of the analytical system throughout the sequence. To further maximize group discrimination and identify differential metabolites, a supervised orthogonal partial least squares discriminant analysis (OPLS-DA) was conducted. The OPLS-DA score plots revealed significant separation between the groups (Figure 6B,C), confirming distinct metabolic profiles across the ND, HFD, and AM groups. Potential biomarkers were identified based on the criteria of Variable Importance in Projection (VIP) > 1, Fold Change (FC) ≥ 1.5 or ≤0.67, and *p* < 0.05. Volcano plots revealed that a total of 418 metabolites were significantly altered in the HFD group relative to the ND group. Furthermore, 214 differential metabolites were identified in the AM intervention compared with the HFD-fed mice (Figure 6D,E). Among these, 21 key metabolites were identified as potential biomarkers of AM intervention (Table S2). These metabolites exhibited significant dysregulation in the HFD-induced MASLD mice but were remarkably reversed toward the trend of normal levels.

To further characterize the specific metabolic variations and the regulatory impact of AM, hierarchical clustering analysis (HCA) was performed on the identified differential metabolites. As illustrated in the heatmap (Figure 6F), clear clustering of metabolic signatures was observed among the ND, HFD, and AM-treated groups, which was consistent with the PCA results. Compared with the ND group, the HFD group exhibited a significant decrease (*p* < 0.05) in the levels of several critical lipid species, including various lysophospholipids (e.g., LysoPC (18:1), LysoPC (18:0), LysoPE (20:1), LysoPE (18:2)), polyunsaturated fatty acids (e.g., 11,14,17-eicosatrienoic acid, oleic acid), and amino acid derivatives (e.g., betaine, L-phenylalanine). Notably, AM supplementation remarkably reversed these HFD-induced alterations, effectively restoring these metabolites toward levels observed in the ND group. Conversely, specific metabolites such as LysoPE (22:6), dihydromuronic acid, and taurocholic acid were significantly elevated in the HFD-induced MASLD mice but were markedly attenuated following AM intervention. These findings suggest that AM exerts a potent regulatory effect on hepatic metabolic disturbances, particularly in ameliorating lipid dyshomeostasis. To elucidate the biological significance of these metabolic shifts, KEGG pathway enrichment analysis was conducted. As presented in Figure 4G, the differential metabolites were primarily enriched in pathways including glycerophospholipid metabolism, biosynthesis of unsaturated fatty acids, arachidonic acid metabolism, primary bile acid biosynthesis, and one-carbon metabolism. Among these, glycerophospholipid metabolism exhibited the highest pathway impact, indicating that membrane lipid remodeling and phospholipid turnover are central to the metabolic regulation mediated by AM. Furthermore, the significant alterations in fatty acid and bile acid biosynthetic pathways suggest that AM treatment improves hepatic lipid utilization and restores systemic energy homeostasis. Taken together, these results suggest that AM intervention effectively mitigates MASLD by reprogramming key lipid metabolism pathways.

## 3. Discussion

As MASLD becomes a leading global cause of chronic liver disease, there is an urgent need for effective multi-target therapies [22]. *Astragalus membranaceus*, a dual-purpose medicinal and edible herb, is renowned for its anti-inflammatory and antioxidant activities [23]. However, the mechanisms underlying its effects on hepatic steatosis are not yet fully understood. This study utilized an integrative systems biology framework—incorporating network pharmacology, metabolomics, and microbiota profiling—to investigate the therapeutic basis of AM against HFD-induced MASLD. This study is primarily designed to evaluate early-stage hepatic alterations and preventive effects rather than advanced disease progression. Within this context, our findings suggest that AM may exert hepatoprotective effects through the coordinated modulation of hepatic metabolism, inflammatory responses, and gut microbial homeostasis, providing a systemic perspective on its potential efficacy.

In this study, a total of 37 major chemical constituents of AM were identified using HPLC-Q-Exactive Orbitrap MS analysis, predominantly flavonoids, triterpenoid saponins, alkaloids, organic acids, and phenylpropanoids, consistent with previous Phytochemical studies [24,25]. Among these, flavonoids such as isorhamnetin, ononin, and rhamnocitrin were the major bioactive components, which have been widely reported to exert antioxidant, anti-inflammatory, and lipid-regulatory activities [26,27,28]. In addition to flavonoids, triterpenoid saponins—particularly astragalosides—represent another important class of bioactive constituents in AM. These compounds have been reported to exert multiple hepatoprotective effects, including the attenuation of oxidative stress, suppression of inflammatory signaling pathways, and modulation of lipid metabolism [29]. Previous studies have demonstrated that astragalosides can regulate key metabolic pathways involved in hepatic lipid homeostasis and improve liver injury in metabolic disease models [30]. Therefore, the presence of triterpenoid saponins in AM may also contribute synergistically to the amelioration of hepatic steatosis and inflammatory responses observed in MASLD. It should be noted that the identified compounds mainly represent prototypic constituents of AM, while their in vivo metabolites were not fully captured in the present analysis. Since many phytochemicals undergo extensive biotransformation after absorption, further studies integrating metabolomics and pharmacokinetic analyses are warranted to elucidate the metabolic fate and biological contributions of these compounds in vivo.

Based on the “disease–gene–target–drug” framework, network pharmacology provides a systemic perspective for evaluating the multi-target effects of botanical interventions [31]. In this study, network analysis identified several hub proteins targeted by AM-derived compounds, including AKT1, TNF, TP53, EGFR, and MAPK1—key regulators of lipid metabolism, insulin signaling, and inflammatory cascades. KEGG enrichment analysis further underscored the involvement of the PI3K–Akt, TNF, and AGE–RAGE signaling pathways [32,33]. Specifically, the PI3K–Akt pathway is essential for maintaining the equilibrium between lipogenesis and fatty acid β-oxidation [34], while the TNF and AGE–RAGE pathways are primary drivers of hepatic oxidative stress and inflammation [35]. These findings suggest that AM exerts pleiotropic effects by modulating interconnected signaling networks rather than a single target, consistent with the holistic therapeutic paradigm of traditional Chinese medicine [36]. Molecular docking further validated these in silico predictions, suggesting that key constituents, such as isorhamnetin and calycosin, exhibit high binding affinities for AKT1 and TNF. The formation of stable hydrogen bonds and hydrophobic interactions suggests that these compounds may directly inhibit pathological signaling, providing a robust structural basis for the hepatoprotective efficacy of AM in MASLD. The predicted targets identified through network pharmacology and molecular docking require further experimental validation, such as Western blot analysis of key signaling pathways including AKT, EGFR, and NF-κB.

This study also observed that AM intervention significantly suppressed HFD-induced weight gain and reduced serum ALT and AST levels, demonstrating a robust hepatoprotective effect. These biochemical improvements were consistent with histopathological observations, where HE staining showed a marked reduction in intrahepatic fat vacuoles. These findings indicate that AM effectively mitigates lipid accumulation and preserves hepatocellular integrity, preventing the progression of MASLD. The therapeutic efficacy of AM is further attributed to its ability to modulate oxidative stress and inflammation [37]. By reducing MDA levels and restoring SOD activity, AM effectively re-establishes redox homeostasis in the liver. Furthermore, the significant downregulation of pro-inflammatory cytokines (IL-1β and IL-6) suggests that AM interrupts the inflammatory cascade associated with lipotoxicity. Collectively, these results confirm that AM protects against MASLD by concurrently inhibiting oxidative damage and the inflammatory response. Although statistically significant differences were observed for several parameters, the relatively small sample size represents a limitation of the present study. Future investigations with larger cohorts (e.g., *n* ≥ 10–12 per group) will be necessary to further confirm the robustness of the observed effects.

Accumulating evidence indicates that gut microbiota dysbiosis plays a critical role in MASLD development via altered energy harvest, impaired intestinal barrier function, and endotoxin-mediated inflammation [38,39]. In this study, AM markedly reshaped the gut microbial community disrupted by HFD feeding. AM treatment restored microbial richness and diversity and shifted the overall microbial structure toward that of normal controls. Notably, AM reduced the elevated Firmicutes/Bacteroidetes ratio induced by HFD, a characteristic feature of obesity-related metabolic dysfunction [40]. At the genus level, AM increased beneficial taxa such as *Akkermansia*, *Bacteroides*, and *Faecalibacterium*, which are known producers of SCFAs [41,42]. SCFAs play essential roles in maintaining intestinal barrier integrity, enhancing hepatic β-oxidation, and suppressing systemic inflammation through the gut–liver axis [43]. Conversely, AM reduced potentially pathogenic genera associated with endotoxemia, insulin resistance, and hepatic inflammation. LEfSe analysis further identified *Akkermansia* and *Bacteroides* as key microbial biomarkers in the AM-treated group, underscoring their contribution to AM-mediated metabolic benefits [44]. Taken together, these findings suggest that AM exerts a prebiotic-like effect by restoring gut microbial balance and enhancing SCFAs-mediated signaling. Therefore, modulation of SCFAs production may contribute to the attenuation of hepatic inflammation and metabolic dysfunction through regulation of the gut–liver axis. This mechanism is consistent with the observed improvements in metabolic, biochemical, and histological outcomes in the present study [45]. Although changes in SCFA-producing bacterial taxa were observed, SCFA concentrations were not directly measured. Future studies integrating targeted metabolite quantification and functional assays will be required to validate the proposed gut–liver axis mechanism.

Metabolomic analysis offered additional mechanistic insight into the metabolic reprogramming induced by AM [46]. HFD feeding resulted in profound disturbances in systemic metabolism, whereas AM treatment significantly restored metabolic profiles toward those of normal controls. Pathway enrichment analysis identified glycerophospholipid metabolism, sphingolipid metabolism, bile acid biosynthesis, tryptophan metabolism, and glutathione metabolism as key pathways modulated by AM. The normalization of glycerophospholipid and sphingolipid metabolism suggests reduced lipid accumulation and membrane oxidative damage [47]. Improved bile acid metabolism indicates enhanced cholesterol homeostasis and hepatic detoxification capacity, while upregulated glutathione metabolism reflects strengthened antioxidant defenses [48]. Collectively, these metabolic adaptations highlight the role of AM in restoring hepatic metabolic homeostasis and mitigating oxidative stress. Notably, disturbances in hepatic lipid metabolism are often associated with systemic hyperlipidemia, which exacerbates metabolic dysfunction and liver injury. Previous studies have shown that AM exerts lipid-lowering effects by regulating lipid metabolism pathways. For instance, Wang et al. reported that AM alleviates high-fat-diet-induced hyperlipidemia by modulating lipid metabolism-related signaling pathways, thereby reducing serum lipid levels and improving hepatic lipid homeostasis [49]. Similarly, Yang et al. demonstrated that AM extracts exhibit lipid-lowering activity through multi-target regulation of lipid metabolism and oxidative stress pathways [50]. These findings are consistent with our metabolomic results, suggesting that AM may ameliorate MASLD partly through regulating lipid metabolism and improving hyperlipidemia-related metabolic disturbances.

## 4. Materials and Methods

### 4.1. Materials and Reagents

AM was supplied by Beiyue Shenqi Co., Ltd. (Datong, Shanxi, China). Additional LC–MS grade reagents, including acetonitrile, methanol, and formic acid, were purchased from Merck KGaA (Darmstadt, Germany). Both the standard diet and high-fat diet were provided by Beijing Speifu Biotechnology Co., Ltd. (Beijing, China). Reference standards, including calycosin-7-glucoside, quercetin, calycosin, astragaloside I, astragaloside II, astragaloside III, isomucronulatol and isoastragaloside I were obtained from Chengdu Lemeitian Pharmaceutical Technology Co., Ltd. (Chengdu, China). Commercial assay kits for determining alanine aminotransferase (ALT) and aspartate aminotransferase (AST) were purchased from Nanjing Jiancheng Bioengineering Institute (Nanjing, Jiangsu, China). ELISA kits used to quantify tumor necrosis factor-α (TNF-α), interleukin-1β (IL-1β), and interleukin-6 (IL-6) were acquired from Shanghai Enzyme-linked Biotechnology Co., Ltd. (Shanghai, China). A 4% tissue fixation solution (paraformaldehyde) was supplied by Beijing Biorigin Biotechnology Co., Ltd. (Beijing, China).

### 4.2. AM Sample Preparation and Chemical Composition Testing

The AM was prepared as previously described [51]. In brief, dried roots of AM were subjected to extraction using 50% fermented ethanol solution under high-temperature conditions at 80 °C for approximately 4 h. The resulting extract was subsequently concentrated under reduced pressure using rotary evaporation. The concentrated solution was freeze-dried to obtain AM powder. For subsequent experiments and qualitative analysis, the lyophilized powder was re-dissolved in phosphate-buffered saline (PBS) and stored at −20 °C until use. Chemical profiling of AM was carried out using an ultra-high-performance liquid chromatography system coupled with Q-Exactive Orbitrap high-resolution mass spectrometry (UHPLC-Q Exactive Orbitrap HRMS, Thermo Fisher Scientific, Milford, MA, USA). Chromatographic separation was performed on a Waters BEH T3 column (2.1 × 100 mm, 1.7 μm). The flow rate was maintained at 0.3 mL/min, the injection volume was set at 5 μL, and the column temperature was controlled at 45 °C. The mobile phase consisted of acetonitrile (solvent A) and purified water (solvent B). Gradient elution was applied as follows: 0–2 min, 96% B; 2–5 min, 96–70% B; 5–7 min, 70% B; 7–15 min, 70–52% B; 15–20 min, 52–20% B; 20–25 min, 20–5% B; and 25–30 min, 5–96% B.

Mass spectrometric detection was performed in both positive and negative ionization modes, with a scanning range of *m*/*z* 50–1050 under full scan conditions. Instrument parameters were optimized as follows: Fourier transform resolution of 70,000 for MS1 and 17,500 for dd/MS2; automatic gain control (AGC) target values of 2 × 10^5^ for MS1 and 1 × 10^5^ for dd/MS2; capillary temperature set to 320 °C; probe heater temperature maintained at 400 °C; spray voltages of +3.5 kV and −3.0 kV for positive and negative modes, respectively. The sheath gas and auxiliary gas flow rates were adjusted to 35 and 10 arbitrary units, respectively. All raw data were processed using Xcalibur software (version 4.1).

### 4.3. Data Processing and Compound Identification

Raw data were processed using Compound Discoverer 4.0 software (ThermoFisher, Milford, MA, USA). The detected high-resolution precursor ions (with diverse adducts) were compared with the Orbitrap Traditional Chinese Medicine product library to formulate a preliminary roster of putative compounds. Candidate constituents were further screened according to their chemical class and relevance to AM, and MS/MS fragment ions were extracted and inspected in TraceFinder 5.2 (ThermoFisher) to confirm or refine the assignments by comparison with previously reported fragmentation patterns in the literature and, when available, authenticated reference standards.

### 4.4. Network Pharmacology and Molecular Docking Study

The potential targets of AM components were predicted via Swiss Target Prediction. The term “MASLD” was retrieved from the DisGeNET (https://www.disgenet.org/) and GeneCards database (https://www.genecards.org/) to acquire disease targets. An intersection analysis was performed between the disease targets of MASLD and the potential targets of AM components via the Jvenn platform (http://jvenn.toulouse.inra.fr, version 1.5.0). The obtained data was employed to establish a protein–protein interaction (PPI) network within the STRING 11.5 database (https://cn.string-db.org/), with “Homo sapiens” designated as the organism and a confidence score of ≥0.7. Subsequently, the “Compounds-Effective Targets-Disease” network and core target PPI network were constructed using Cytoscape 3.9.1 software, with screening parameters set as “degree > median, median centrality > median, centrality proximity > median”. The core targets identified were further analyzed using KEGG and GO databases. The Cytohubba plug-in in Cytoscape 3.9.1 was used to screen key active compounds and key targets. For molecular docking, the three-dimensional structures of the target proteins were acquired from the RCSB Protein Data Bank (http://www.rcsb.org/), whereas the compound structures were retrieved from the PubChem database. Ligand and protein preparation and docking simulations were performed using Chem3D 19.0, PyMOL 1.7.4.5, AutoDock Tools 1.5.6, and AutoDock Vina 1.1.2.

### 4.5. Animal Models and Ethics

All experimental procedures involving animals complied with institutional guidelines for animal care and were authorized by the Animal Ethics Committee of the First Affiliated Hospital, Zhejiang University School of Medicine (Approval No. 20231466). Male C57BL/6 mice (5 weeks old, 20 ± 2 g) were purchased from GemPharmatech Co., Ltd. (Nanjing, China). The animals were maintained in the specific pathogen-free (SPF) facility under controlled environmental conditions, including a temperature of 22–23 °C, relative humidity of 40–45%, and a 12 h light/dark cycle. After the acclimatization period, 24 male C57BL/6 mice were randomly assigned to 4 experimental groups (*n* = 6) using a random number generator as follows: (1) normal dietgroup (ND, 16% calories from fat), (2) high fat diet group (HFD, 60% calories from fat), (3) HFD + 100 mg/kg AM extract group (AM), (4) HFD + 10 mg/kg Simvastatin group (Sim). The oral gavage of an AM dose of 100 mg/kg was chosen in accordance with previous research [51]. All mice were housed in a controlled environment in a specific pathogen-free animal facility with free access to water. After 4 weeks of HFD-induced MASLD, all samples were dissolved in phosphate-buffered saline (PBS) and orally administered on a daily basis for a duration of 8 weeks. Body weights and food intake were measured on a weekly basis. At the conclusion of the experimental period, all mice were subjected to a 12 h fasting period and subsequently anesthetized using a mixture of Zoletil (50 mg/mL, Virbac, Carros, France) and Rompun (23.32 mg/mL, Bayer, Leverkusen, Germany) (at a ratio of 1:3. Fecal samples from the mice were collected under sterile conditions and stored in a −80 °C refrigerator after rapid freezing with liquid nitrogen for the analysis of intestinal flora diversity. Blood specimens were obtained and centrifuged to collect plasma for subsequent testing. The livers were excised, weighed, and then either immersed in 4% paraformaldehyde or stored at −80 °C after being flash-frozen in liquid nitrogen.

### 4.6. Histological Examination

Histopathological analysis was performed as previously described [20]. Liver samples were immersed in 4% paraformaldehyde solution for 48 h and embedded in paraffin wax before sectioning for histological examination using Haematoxylin and eosin (H&E) staining. Subsequently, the stained sections were mounted using neutral resin. Images were captured under a microscope at a magnification of 400×, and histological evaluation was conducted by an investigator who was blinded to the group allocation.

### 4.7. Serum Biochemical Measurements

Serum samples were retrieved from a −80 °C freezer and subjected to slow thawing at 4 °C prior to analysis. Serum levels of aspartate aminotransferase (AST) and alanine aminotransferase (ALT) were determined using commercially available assay kits (Ningbo Saike Biological Technology Co., Ltd., Ningbo, China) according to the manufacturer’s instructions. The concentrations of TNF-α, IL-1β, and IL-6 in serum were quantified using ELISA kits obtained from Shanghai Enzyme-linked Biotechnology Co., Ltd. (Shanghai, China), following the manufacturer’s protocols.

### 4.8. Liver Metabolomics Analysis

#### 4.8.1. Sample Preparation

For hepatic metabolomics analysis, approximately 25 mg of liver tissue was accurately weighed and transferred into pre-cooled centrifuge tubes. Subsequently, 1000 μL of pre-chilled 80% (*v*/*v*) methanol was added to each sample to precipitate proteins and quench enzymatic activity. The mixture was subjected to two rapid freeze–thaw cycles using liquid nitrogen to enhance metabolite extraction efficiency. Samples were then incubated at −20 °C for approximately 1 h, followed by centrifugation to collect the supernatant. The supernatant was dried under a gentle nitrogen stream to minimize oxidative degradation. The dried residue was reconstituted in 100 μL of acetonitrile–water (1:1, *v*/*v*), vortex-mixed thoroughly, and used as the test solution. Quality control (QC) samples were prepared by pooling 20 μL aliquots from each individual sample to represent the overall metabolic composition of the dataset. Serum samples were processed and analyzed under identical experimental conditions to ensure methodological consistency.

#### 4.8.2. UPLC–Q-TOF/MS Conditions

Metabolomic profiling was performed using an ultra-performance liquid chromatography system coupled with quadrupole time-of-flight mass spectrometry (UPLC–Q-TOF/MS). Chromatographic analysis was carried out on a Waters Acquity UPLC platform (Waters Corporation, Milford, MA, USA) fitted with an Acquity UPLC HSS T3 column (100 mm × 2.1 mm, 1.8 μm), with the column temperature maintained at 40 °C. The mobile phase consisted of acetonitrile (solvent A) and water containing 0.1% (*v*/*v*) formic acid (solvent B), and was delivered at a flow rate of 0.4 mL/min. The sample injection volume was 2 μL. Gradient elution was programmed as follows: 2% A from 0 to 0.25 min, 2–90% A from 0.25 to 10 min, 98% A from 10 to 13 min, 98–2% A from 13 to 13.1 min, followed by 2% A from 13.1 to 15 min to re-establish column equilibrium.

Mass spectrometric analysis was conducted using a Waters Xevo G2-XS Q/TOF mass spectrometer (Waters Corporation, Milford, MA, USA) equipped with an electrospray ionization (ESI) source operated in the negative ionization mode. Instrument parameters were optimized as follows: capillary voltage −3.0 kV, cone voltage −30 V, source temperature 120 °C, desolvation temperature 550 °C, desolvation gas flow 900 L/h, and cone gas flow 50 L/h. The mass detection range was set to *m*/*z* 50–1200. To maintain accurate mass calibration during acquisition, leucine enkephalin was continuously introduced as a lock-mass reference compound. Data acquisition was performed in data-dependent acquisition (DDA) mode, applying a low collision energy of 5 eV and a high collision energy ramp of 15–55 eV, allowing simultaneous recording of precursor ions and their corresponding fragment ions.

#### 4.8.3. Data Processing and Statistical Analysis

Raw mass spectrometric datasets were initially handled using MassLynx 4.2, after which the processed files were imported into Progenesis QI for feature extraction, retention time alignment, and signal normalization. Metabolite annotation was achieved by comparing accurate mass measurements together with MS/MS fragmentation information against entries in the Human Metabolome Database (HMDB) as well as a customized Progenesis QI spectral library. To evaluate analytical stability and maintain data reliability, metabolites showing a relative standard deviation (RSD) above 30% in quality control (QC) samples were removed from subsequent analysis. The filtered dataset was then normalized based on the total ion peak area, which minimizes systematic fluctuations in signal intensity resulting from variations in sample preparation procedures, injection volumes, and instrumental response, thereby improving comparability among samples. Multivariate statistical modeling was conducted using SIMCA software (version 14.1). Orthogonal partial least squares discriminant analysis (OPLS-DA) was employed to visualize differences among experimental groups and to screen metabolites contributing to group discrimination. The variable importance in projection (VIP) value was calculated to estimate the influence of each metabolite within the model. Metabolites satisfying the criteria of VIP > 1.0 together with a Student’s *t*-test *p* value < 0.05 were considered significantly altered. Pathway enrichment analysis of significantly altered metabolites was performed using MetaboAnalyst 6.0 to identify metabolic pathways associated with the observed biochemical changes.

### 4.9. 16S rRNA Gene Amplicon Sequencing of Faecal Microbiota

Stool samples were immediately snap-frozen in liquid nitrogen and stored at −80 °C until further analysis to preserve microbial composition and DNA integrity. Total genomic DNA was extracted from fecal samples using the cetyltrimethylammonium bromide/sodium dodecyl sulfate (CTAB/SDS) method following the manufacturer’s recommended procedures. The quality and integrity of DNA were evaluated via agarose gel electrophoresis, and the DNA concentration was ascertained prior to polymerase chain reaction (PCR) amplification. The V3–V4 hypervariable region of the bacterial 16S rRNA gene was amplified using the universal primer pair (forward: CCTAYGGGRBGCASCAG; reverse: GGACTACNNGGGTATCTAAT). PCR products were purified and quantified to ensure appropriate fragment size and concentration before library construction. Sequencing libraries were prepared according to standard Illumina protocols and sequenced on an Illumina NovaSeq 6000 platform (Illumina, San Diego, CA, USA) using paired-end 250 bp reads (PE250). Raw paired-end reads were subjected to stringent quality control to remove low-quality sequences and technical artifacts. Adapter trimming and quality filtering were performed using Trimmomatic (v0.33) and Cutadapt (v1.9.1). High-quality paired-end reads were merged, and additional filtering was conducted using USEARCH (v10.0). Chimeric sequences generated during PCR amplification were identified and removed using UCHIME (v4.2) to minimize artificial inflation of microbial diversity. The remaining high-quality sequences were clustered into operational taxonomic units (OTUs) at a 97% sequence similarity threshold using USEARCH (v10.0). Representative OTU sequences were taxonomically annotated against the SILVA reference database (http://www.arb-silva.de) using a Naive Bayes classifier (QL Server Analysis Services, Redmond, WA, USA) to obtain taxonomic classification information from phylum to genus levels. An OTU abundance table was generated, and the relative abundance of OTUs across samples was calculated and used for subsequent microbial community composition, diversity, and comparative analyses.

### 4.10. Statistical Analysis

Statistical evaluation was carried out using both Student’s *t*-test and the Mann–Whitney U test. A *p*-value below 0.05 was considered indicative of statistical significance (ns, not significant; * *p* < 0.05; ** *p* < 0.01; *** *p* < 0.001; **** *p* < 0.0001). For clustering analysis, the TimeSeriesExperiment and Mfuzz packages implemented in the R (version 4.1) statistical environment were applied. Experimental results are presented as mean ± SEM, and graphical visualization and statistical calculations were conducted using GraphPad Prism version 9.0 (GraphPad Software, San Diego, CA, USA). In addition to conventional statistical testing, effect sizes (Cohen’s *d*) were calculated to quantify the magnitude of differences between groups. Cohen’s *d* was computed for pairwise comparisons (ND vs. HFD, HFD vs. AM, and HFD vs. SIM) using pooled standard deviations. Furthermore, post hoc statistical power analysis was performed based on a two-tailed independent samples *t*-test (α = 0.05) using the observed effect sizes and sample size (*n* = 6 per group). Effect sizes were interpreted as small (0.2), medium (0.5), and large (0.8), while values exceeding 2.0 were considered indicative of very large biological effects.

## 5. Conclusions

In conclusion, this study demonstrates that AM may effectively alleviate hepatic steatosis and inflammation in MASLD through a multi-component, multi-target, and multi-pathway regulatory mechanism involving hepatic metabolic reprogramming and gut microbiota modulation. Nevertheless, the use of non-targeted metabolomics and 16S rRNA sequencing limits functional resolution. Future studies integrating metagenomic sequencing and targeted metabolomics are warranted to further elucidate the molecular crosstalk between the gut and liver under AM intervention.

## Figures and Tables

**Table 1:** The chemical components identification of AM solution and serum sample based on UPLC-Q-orbitrap-MS/MS.

No.	Name	Formula	RT	Ion Mode	Calc *m*/*z*	Exact *m*/*z*	Error (ppm)	Fragment Ions	Class
1	Sucrose	C_12_H_22_O_11_	1.35	[M − H]^−^	341.1083	341.1088	−1.47	341, 179, 119, 101, 89	Saccharides
2	Adenine	C_5_H_5_N_5_	1.92	[M − H]^−^	134.0469	134.0472	−2.24	134, 107, 92, 65	Nucleobases
3	Citric Acid	C_6_H_8_O_7_	2.11	[M − H]^−^	191.0194	191.0197	−1.57	129, 111, 87, 85	Organic Acids
4	Uridine	C_9_H_12_N_2_O_6_	2.29	[M − H]^−^	243.0616	243.0623	−2.88	243, 152, 130, 82	Nucleobases
5	L-Phenylalanine	C_9_H_11_NO_2_	4.89	[M − H]^−^	164.0713	164.0717	−2.44	164, 147, 103, 72	Amino Acids
6 ^S^	L-Tryptophan	C_11_H_12_N_2_O_2_	8.04	[M − H]^−^	203.0820	203.0828	−3.94	142, 116	Amino Acids
7	Calycosin-7-glucoside *	C_22_H_22_O_10_	11.48	[M + H]^+^	447.1285	447.1300	−0.16	285, 270	Flavonoids
8	Isomucronulatol-7,2′-Di-O-Glucoside	C_29_H_38_O_15_	12.34	[M − H]^−^	625.2095	625.2138	−6.88	579, 417, 271	Flavonoids
9 ^S^	Iristectorin A	C_23_H_24_O_12_	12.46	[M + FA − H]^−^	491.1189	491.1195	−1.22	491, 329, 313, 298	Flavonoids
10	6″-O-Acetylglycitin	C_24_H_24_O_11_	13.39	[M + H]^+^	489.1395	489.1400	0.74	489, 185, 270, 137	Flavonoids
11	Baicalin	C_21_H_18_O_11_	13.49	[M − H]^−^	445.0767	445.0761	1.35	269, 241, 169, 113	Flavonoids
12 ^S^	Ononin	C_22_H_22_O_9_	13.58	[M + FA − H]^−^	475.1242	475.1246	−0.84	475, 267, 251	Flavonoids
13	10-Hydroxy-3,9-Dimethoxypterocarpan	C_17_H_16_O_5_	14.04	[M + H]^+^	301.1076	301.1100	1.83	167, 152, 134, 106	Flavonoids
14	Isomucronulatol-7-O-Glc	C_23_H_28_O_10_	14.23	[M + FA − H]^−^	509.1661	509.1664	−0.59	301, 286, 270, 269	Flavonoids
15 ^S^	Isorhamnetin	C_16_H_12_O_7_	14.53	[M − H]^−^	315.0501	315.0510	−2.86	315, 300, 271, 151	Flavonoids
16	Quercetin *	C_15_H_10_O_7_	14.53	[M + H]^+^	303.0509	303.0500	3.2	303, 179, 153, 137	Flavonoids
17	Calycosin *	C_16_H_12_O_5_	14.58	[M − H]^−^	283.0613	283.0600	0.35	268, 211, 184, 135	Flavonoids
18	Dihydroxy-Dimethoxyisoflavone	C_17_H_14_O_6_	14.78	[M + H]^+^	315.0867	315.0900	1.24	315, 300, 243, 107	Flavonoids
19	Rhamnocitrin or isomer	C_16_H_12_O_6_	15.42	[M − H]^−^	299.0553	299.0561	−2.68	284, 256, 151, 107	Flavonoids
20 ^S^	Astragaloside IV *	C_41_H_68_O_14_	15.87	[M + FA − H]^−^	829.4581	829.4591	−1.21	829, 783, 489, 179	Triterpenoid Saponins
21 ^S^	Rhamnocitrin	C_16_H_12_O_6_	15.90	[M − H]^−^	299.0554	299.0561	−2.34	284, 227, 199, 107	Flavonoids
22	Astragaloside III *	C_41_H_68_O_14_	15.98	[M + FA − H]^−^	829.4587	829.4600	−0.49	829, 783, 489	Triterpenoid saponins
23	Astragaloside A	C_41_H_68_O_14_	16.00	[M + FA − H]^−^	829.4581	829.4591	−1.21	829, 783, 489, 179	Triterpenoid Saponins
24 ^S^	Formononetin	C_16_H_12_O_4_	16.57	[M − H]^−^	267.0655	267.0663	−3.00	252, 223, 195, 132	Flavonoids
25	Astragaloside II *	C_43_H_70_O_15_	16.65	[M + FA − H]^−^	871.4673	871.4697	−2.75	871, 825, 765	Triterpenoid Saponins
26	7-Hydroxy-6,4′ -Dimethoxyisoflavone	C_17_H_14_O_5_	16.76	[M + H]^+^	299.0918	299.0900	1.34	299, 284, 256	Flavonoids
27	Soyasaponin Bb	C_48_H_78_O_18_	16.84	[M + FA − H]^−^	987.5138	987.5121	1.72	987, 941	Triterpenoid Saponins
28	Isomucronulatol *	C_17_H_18_O_5_	16.95	[M + H]^+^	303.1229	303.1200	0.66	152, 167, 161, 133, 123, 95	Flavonoids
29	Malonyl-saikosaponin A	C_45_H_70_O_16_	17.14	[M − H]^−^	865.4579	865.4554	2.89	85, 821, 614,	Triterpenoid Saponins
30 ^S^	Isoastragaloside II	C_43_H_70_O_15_	17.16	[M + FA − H]^−^	871.4673	871.4697	−2.75	871, 825, 765	Triterpenoid Saponins
31 ^S^	Biochanin-A	C_16_H_12_O_5_	17.60	[M − H]^−^	283.0604	283.0612	−2.83	268, 239, 163, 110	Flavonoids
32	Biochanin-A or isomer	C_16_H_12_O_5_	17.95	[M − H]^−^	283.0604	283.0612	−2.83	268, 239, 163, 110	Flavonoids
33 ^S^	Isoastragaloside I *	C_45_H_72_O_16_	18.10	[M + FA − H]^−^	913.4778	913.4802	−2.63	913, 867, 807, 161	Triterpenoid Saponins
34	Astragaloside I *	C_45_H_72_O_16_	18.54	[M + FA − H]^−^	913.4778	913.4802	−2.63	913, 867, 807, 161	Triterpenoid Saponins
35	2-[4-(2-Hydroxy-Ethoxy)-1-Isobutyl-1,4,6-Trimethyl-Hept-2-Ynyloxy]-Ethanol	C_18_H_34_O_4_	19.08	[M − H]^−^	313.2377	313.2384	−2.23	295, 277, 201	Sesquiterpenoids
36	Acetylastragaloside I	C_47_H_74_O_17_	20.23	[M + FA − H]^−^	955.4896	955.4908	−1.26	955, 793, 731	Triterpenoid Saponins
37	Hederagenin	C_30_H_48_O_4_	20.51	[M + H]^+^	473.3630	473.3600	0.97	455, 437, 329, 295, 173, 121	Triterpenes

Note: ^S^ Both detected in AM solution and mice serum sample; *: confirmation in comparison with authentic standards; RT: retention time.

## Data Availability

The original contributions presented in this study are included in the article/Supplementary Material. Further inquiries can be directed to the corresponding author.

## Supplementary Materials

The following supporting information can be downloaded at https://www.mdpi.com/article/10.3390/molecules31071120/s1: Figure S1. Predicted potential targets of major active compounds and the construction of the “compound-target” network; Table S1. Molecular docking binding energies (kcal/mol) of major active compounds with key target proteins (EGFR, TP53, STAT1, NFKB1, and TNF); Table S2. Effect sizes (Cohen’ s *d*) and post hoc statistical power for pairwise comparisons between experimental groups; Table S3. Differential hepatic metabolites among distinct groups of mice identified through metabolomics analysis using UHPLC-Tof-MS/MS.
