# Supplementary material for: Integrative Multiomics Analysis Reveals the Ameliorative Effects of Astragalus membranaceus Extract on Metabolic Dysfunction-Associated Steatotic Liver Disease
Source: Molecules. 2026 Mar 28;31(7):1120. doi: 10.3390/molecules31071120 (PMC13074959; doi:10.3390/molecules31071120)
Supplement: Supplementary file 1 [file molecules-31-01120-s001.zip › molecules-4185211-supplementary.pdf]

Figure S1. Predicted potential targets of major active compounds and the construction of the "compound-target" network .

Table S1. Molecular docking binding energies (kcal/mol) of major active compounds with key target proteins (EGFR, TP53, STAT1, NFKB1, and TNF).

Table S2. Effect sizes (Cohen' s *d*) and post hoc statistical power for pairwise comparisons between experimental groups.

Table S3. Differential hepatic metabolites among distinct groups of mice identified through metabolomics analysis using UHPLC-Tof-MS/MS.

Figure S1.

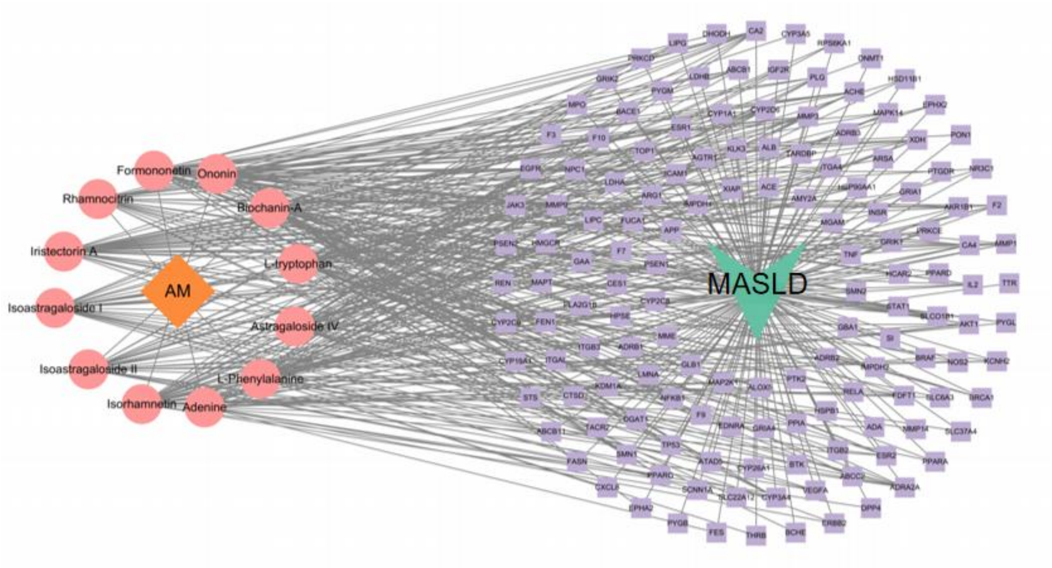
Formononetin
Ononin
Rhamnocitrin
Biochanin-A
Iristectorin A
L-tryptophan
AM
Isoastragaloside I
Astragaloside IV
Isoastragaloside II
L-Phenylalanine
Isorhamnetin
Adenine
MASLD

Table S1 Molecular docking binding energies (kcal/mol) of major active compounds with key target proteins (EGFR, TP53, STAT1, NFKB1, and TNF)

**Compounds**	**EGFR**	**TP53**	**STAT1**	**NFKB1**	**TNF**
Adenine	-5.5	-4.5	-4.3	-5	-5.7
Astragaloside IV	-7.5	-6.9	-7.0	-6.7	-7.4
Biochanin-A	-8.5	-6.6	-7.3	-6.4	-8.4
Formononetin	-8.3	-6.0	-6.2	-6.3	-8.1
Iristectorin A	-8.6	-7.1	-7.7	-6.9	-9.2
Isoastragaloside I	-6.8	-6.7	-7.2	-6.7	-7.3
Isoastragaloside II	-7.2	-6.7	-7.2	-6.6	-7.1
Isorhamnetin	-8.7	-6.7	-8.7	-7.3	-8.9
L-Phenylalanine	-5.6	-5.0	-4.1	-4.5	-5.4
L-tryptophan	-6.7	-4.9	-4.5	-4.8	-6.5
Ononin	-7.8	-7.2	-6.8	-7.4	-9.3
Rhamnocitrin	-8.3	-6.1	-7.9	-7.2	-8.8

Table S2. Effect sizes (Cohen’ s d) and post hoc statistical power for pairwise comparisons between experimental groups.

**Marker**	**ND vs HFD (*d*)**	**Power**	**HFD vs AM (*d*)**	**Power**	**HFD vs SIM (*d*)**	**Power**
ALT	4.48	1	2.3	0.95	2.76	0.99
AST	4.57	1	2.22	0.93	2.8	0.99
MDA	5.48	1	2.11	0.91	2.4	0.96
SOD	4.27	1	3.54	1.00	3.57	1.00
IL-1	3.55	1	2.00	0.88	2.22	0.93
IL-6	3.56	1	2.07	0.9	2.09	0.90

Data are presented as Cohen’s *d* values and corresponding statistical power (two-tailed, $\alpha = 0.05$, $n = 6$ per group).

Table S3. Differential hepatic metabolites among distinct groups of mice identified through metabolomics analysis using UHPLC-Tof-MS/MS.

No.	Metabolite name	Rt(min)	Adduct	Mz	HFD/ND			AM/HFD		
					VIP	P	FC	VIP	P	FC
1	1-Palmitoylphosphatidylcholine	14.74	$[M-H]^-$	494.32507	1.38	0.0112	0.15	1.37722	0.0008	3.73
2	LysoPE(20:1(11Z)/0:0)	13.87	[M-H]-	506.32501	1.28	0.0045	0.24	1.2562	0.0075	2.67
3	Aleuretic Acid	14.27	$[M-H]^-$	303.22638	1.53	0.0459	0.25	1.53354	0.0000	3.17
4	11.14.17-eicosatrienoic acid	16.56	$[M-H]^-$	305.24756	1.26	0.0262	0.28	1.26426	0.0085	1.97
5	LysoPE(0:0/18:2(9Z,12Z))	10.72	$[M-H]^-$	476.27731	1.23	0.0017	0.35	1.23318	0.0135	2.01
6	LysoPE(20:0/0:0)	12.55	$[M+H]^+$	510.35294	1.49	0.0380	0.37	1.49095	0.0072	2.12
7	Oleic acid	15.82	$[M-H]^-$	281.23788	1.06	0.0353	0.49	1.05954	0.0957	1.43
8	L-(-)-Phenylalanine	2.07	$[M-H]^-$	164.07124	1.02	0.0041	0.54	1.01915	0.1368	1.27
9	LysoPC(18:1(11Z)/0:0)	10.50	$[M+HCOO]^-$	566.33667	1.14	0.0083	0.58	1.13506	0.0251	1.51
10	LysoPE(18:1(11Z)/0:0)	12.25	$[M-H]^-$	478.29413	1.36	0.0257	0.61	1.36391	0.0012	1.98
11	LysoPC(0:0/18:2(9Z,12Z))	10.50	$[M+H]^+$	520.33887	1.30	0.0136	0.62	1.30055	0.0244	1.46
12	Indoline	2.23	$[M+H]^+$	120.08054	1.13	0.0208	0.64	1.13291	0.0719	1.29
13	LysoPC(18:0/0:0)	13.21	$[M+CH3COO]^-$	568.36157	1.04	0.0055	0.67	1.04181	0.0565	1.39
14	1-palmitoyl-2-oleoyl-sn-glycero-3-phosphocholine	15.82	$[M+H]^+$	760.58557	1.01	0.0097	0.74	1.00908	0.2190	1.97
15	LysoPE(20:5(5Z,8Z,11Z,14Z,17Z)/0:0)	9.74	$[M+H]^+$	500.27615	1.41	0.0352	0.85	1.40659	0.0167	3.04
16	2-Phenylacetamide	1.24	$[M+H]^+$	136.07487	1.29	0.0253	0.86	1.13354	0.5625	1.06
17	LysoPE(18:1(9Z)/0:0)	11.88	$[M-H]^-$	478.29434	1.21	0.0114	0.90	1.21458	0.0071	1.20
18	Betaine	23.92	[M+H]+	118.08556	0.92	0.0383	1.30	1.3252	0.1148	0.54

19	LysoPE(22:6(4Z,7Z,10Z,13Z,16Z,19Z)/0:0)	10.43	$[M-H]^-$	524.27655	1.10	0.0475	1.30	1.23259	0.0719	0.84
20	Dihydromuronic acid	15.87	$[M-H]^-$	367.24527	1.08	0.0157	1.40	1.07743	0.0691	0.86
21	Taurocholic acid	7.03	$[M-H]^-$	514.28528	1.20	0.0114	1.59	1.20035	0.0158	0.31
